# Drivers of iPad use by undergraduate medical students: the Technology Acceptance Model perspective

**DOI:** 10.1186/s12909-022-03152-w

**Published:** 2022-02-08

**Authors:** Doan Hoa Do, Sawsen Lakhal, Mikaël Bernier, Jasmine Bisson, Linda Bergeron, Christina St-Onge

**Affiliations:** 1 Faculty of Medicine and Health Sciences, Université de Sherbrooke, 3001 12e Avenue Nord, Sherbrooke, QC J1H 5N4 Canada; 2 Faculty of Education, Université de Sherbrooke, Sherbrooke, Canada; 3 Faculty of Medicine, Université de Montréal, Montréal, Canada

**Keywords:** Mobile technology, Technology Acceptance Model, Predictive modeling, Undergraduate medical education

## Abstract

**Background:**

Medical students need to acquire a continuously growing body of knowledge during their training and throughout their practice. Medical training programs should aim to provide students with the skills to manage this knowledge. Mobile technology, for example, could be a strategy used through training and practice. The objective of this study was to identify drivers of using mobile technology (an iPad) in a UGME preclinical settings and to study the evolution of those drivers over time.

**Methods:**

We solicited all students from two cohorts of a preclinical component of a Canadian UGME program. They were asked to answer two online surveys: one on their first year of study and another on the second year. Surveys were built based on the Technology Acceptance Model (TAM) to which other factors were also added. Data from the two cohorts were combined and analysed with partial least squares structural equation modelling (PLS-SEM) to test two measurement models, one for each year.

**Results:**

We tested fifteen hypotheses on both data sets (first year and second year). Factors that explained the use of an iPad the first year were knowledge, preferences, perceived usefulness and anticipation. In the second year, perceived usefulness, knowledge and satisfaction explained the use of an iPad. Other factors have also significantly, but indirectly influenced the use of the iPad.

**Conclusions:**

We identified factors that influenced the use of an iPad in a preclinical medical program. These factors differed from the first year to the second year in the program. Our results suggest that interventions should be tailored for different point in time to foster the use of an iPad. Further study should investigate how interventions based on these factors may influence implementation of mobile technology to help students acquire ability to navigate efficiently through medical knowledge.

## Background

Medical students are faced with the immense challenge of acquiring and subsequently managing an ever-growing body of knowledge. While there exist multiple platforms that can facilitate the access to this body of knowledge, we have yet to establish how we can foster the use of mobile technology among students. Contributing to student's digital competence will allow training programs to prepare them for their future professional practice.

Keeping current with the latest medical information is a formidable task. Medical knowledge ‘doubling time’ was estimated to be 50 years in 1950, down to seven years in 1980, and further down to 3.5 years in 2010 [1]. In 2014, Alper et al. [2] counted 341 active journals which published monthly an estimated quantity of 7,287 articles interested in primary care. Because of synthesis efforts, by 2004 the Cochrane Collaboration had amassed an impressive 1,837 systematic reviews addressing intervention-related questions and 11,669 abstracts of additional published systematic reviews [3]. Thus, the challenge is immense for medical students who are not only updating new knowledge but are also developing their knowledge base.

Mobile technologies such as smartphones and tablets are useful tools to navigate and manage the massive amount of available medical knowledge, since they permit rapid access to medical information via applications, online databases, library services and the general Web [4]. The utility of mobile technologies extends to the clinical settings since they exist in a portable format and are therefore easy to carry in clinics and on the wards. In clinical settings and pre-clinical settings, mobile technologies are used to access medical information [4–6] but also to perform clinical calculations [7], to take notes [7, 8], to consult images [4, 9], to access one’s schedule and email [6, 8, 10], and in preparation for examination [11].

Mobile technologies are perceived to be easy to use [12], and seem to generate enthusiasm among students [9, 12]. Reported specific advantages of these technologies include reducing paper use [5, 9], not having to transport multiple textbooks [9], a rapid access to pertinent resources [6, 9] and the ability to make use of downtime [6, 10, 13, 14]. As such, mobile technologies have been introduced in many medicine programs [15] as a response to the growth of medical knowledge, and, probably, technological advancements. However, the introduction of mobile technologies is costly, and knowing how to foster acceptance of mobile technologies is important. The Technology Acceptance Model (TAM), for example, has been used to identify the drivers that influence the use of mobile technology and applications [16], and some studies specifically focused on the domain of medicine [17–19].

Several researchers have investigated the acceptance of mobile devices and apps among medical students at different levels of their training [17–20]. Briz-Ponce and colleagues [17, 18] investigated medical students from year 1 to 4, as well as professionals in the medical sector, without distinguishing the results according to the level; Harmon [19] focused on first year students; and Hart and Sutcliffe [20] on clerkship students. Not surprisingly, these authors found different drivers of the acceptance of mobile technologies and apps [17–19]. Other researchers have focused on studying the acceptance of mobile devices and apps longitudinally. For example, Hart and Sutcliffe [20] studied, over six months, the experience of medical students at the beginning of their clerkship using iPads to support their learning in a hospital context. Using questionnaires and interviews, they found that Perceived ease of use and Perceived usefulness improved over time. They also look at positive and negative affect related to the iPad and found no trend of change over time. Students’ level in a curriculum may influence what drivers will be important to the acceptance of technology. Drivers of the acceptance of technology may also evolve over time.

The aforementioned studies inform us on the acceptance of mobile technologies and apps at different levels of the medical curriculum, and most were conducted at one point in time. Yet, there is value in identifying determinants of technology use earlier in the undergraduate medical education curriculum, and also in tracking the fluctuations of determinants over time which contribute to the implementation of more focused interventions. Therefore, the objectives of this study are (1) to determine the factors that influenced the self-reported use of an iPad in a pre-clinical undergraduate medical education curriculum at two different times (first vs. second year), in order to (2) study the evolution of those factors overtime.

## Methods

### Theoretical framework

The TAM explores why potential users of technology are inclined (or not) to adopt said technology. The original TAM model proposes that cognitive response to system design features (i.e. perceived usefulness [the degree to which a person believes that using a particular system will enhance his/her job performance], and perceived ease of use [the degree to which a person believes that using the technology system would be free of effort]) influences affective response (attitudes towards usage), which explains and drives technology system adoption [21]. In a second version of the TAM, Venkatesh and Davis [22] added other variables to the initial model and dropped attitude toward usage. While concluding on the impact of the other variables, they suggested for further research on TAM to refine the model of the determinants [22].

In the field of technology use and acceptance in health professions education, authors have proposed to add different factors or dimensions to the TAM when using the model. For example, 1- related to the cognitive response, Hart and Sutcliffe [20] and Holden & Rada [23] added usability, and Briz-Ponce and colleagues [17, 18] suggested to include anxiety; 2- related to the affective response, Hart and Sutcliffe [20] added positive or negative affect towards the technology and Harmon [19] added perceived enjoyment; and 3- related to other concepts such as individual characteristics, Briz-Ponce and colleagues [17, 18] included self-efficacy.

We chose to integrate cognitive, affective and other factors to the TAM model, as defined above. As cognitive factors, we have considered perceived usefulness, as well as perceived ease of use and usability. This later “double concepts” (i.e., perceived ease of use and usability) was also considered by Holden & Rada [23] who found a better model fit using this double concepts rather than only perceived ease of use. We have also added other user’s cognitive response in the model, namely expectation and anticipation, as suggested by Townsend et al. [24]. These cognitive responses may predict the attitudes toward the technology. In accordance with Townsend et al. [24], we chose to look at experience-based attitudes, such as satisfaction with the technology after its use. As another affective factor, we have included preferences (as suggested by Mang & Wardley [25]) in our model. Finally, we have added an individual characteristic, that is, self-efficacy to the model in our study. Lastly, we have considered variables to document knowledge about the different device applications as we hypothesized it could influence its use.

Researchers have either used behavioural intention or the actual use of a technology when studying the factors that influence its acceptance. Among previous research in medical education, many of them have considered behavioral intention to use technology and searched for its determinants (i.e. [17–19]). Since our study was conducted in an authentic context, that is, the use of an iPad that has already been implemented, we chose to look at the actual use of the technology rather than the behavioural intention.

### Design

We conducted a descriptive longitudinal study design, involving two data collection time points over two years. Ethics approval was obtained at our local institution from the Education - Social Sciences, Research Ethics Board (*Comité d’éthique de la recherche – Éducation et sciences sociales*).

### Context

The Université de Sherbrooke is a French speaking public medical school in Canada. The undergraduate medical education program offers a 4-year curriculum which is divided in preclinical training for the first two years and a half and clinical training for the last year and a half. In 2014, the Faculty of Medicine and Health Sciences (*Faculté de médecine et des sciences de la santé [FMSS]*) initiated a pilot project to evaluate the benefits of transitioning to a digitally oriented learning environment. First and second year medical students were offered the option to purchase an iPad mini and use electronic versions of the education material rather than the traditional paper versions. Based on the positive feedback received, the faculty proceeded with a complete transition of the education material to an electronic format as of fall 2015, with no paper versions being available. Medical textbooks and other references were offered electronically via the library subscription services, totally free of charge for the students. An iPad mini, paid in full by the *Société des médecins de l’Université de Sherbrooke*, was offered to each student of the 2015-2019 and the 2016-2020’s promotions. The devices were set up and administered by the faculty IT Center and came with pre-installed medical applications recommended by the medical librarian; for instance, Medscape^1^ and MedCalx^2^. In addition, information was provided to the students on how to install faculty-wide services; such as Office 365, which includes a Cloud-based storage system (OneDrive), and on how to synchronise their academic calendar and email on their device. A 90-minute training session was given to students on how to download course notes on iBook format, how to navigate through an iBook, how to consult a reference book online on the FMSS library website, how to download PDF documents or online book chapters, how to highlight and take notes in PDF / iBook documents and automatically save their work in the Cloud-based storage system (OneDrive).

### Participants and recruitment

All students newly enrolled in the medicine undergraduate program (falls 2015 and 2016) were invited to participate in the study (2015-2019: 1rst year: *N* = 210, second year: *N* = 214; 2016-2020: 1rst year: *N* = 207, second year: *N* = 204).

### Material

A survey was developed based on the modified version of the TAM proposed by Holden and Rada [23], to which we added items of cognitive responses, affective responses and individual characteristics. Items in the survey were designed to document nine variables (see Table 1). Participants were asked to rate their degree of agreement with each item on a 4-point Likert Scale (from *I strongly disagree* to *I strongly agree*), except for the items of use of iPad, and items of self-efficacy. We measured frequency of using the iPad with a five-point Likert scale (i.e., *Never, Rarely, Sometimes, Often, Very often*). Self-efficacy was measured with a six-point Guttman scale (from *Not at all confident* to *Totally confident*) as proposed by Holden and Rada [23].

**Table 1:** Variables included in the questionnaire with corresponding number of items, category of variables, and scale of measurement

Variables	Number of items	Categories of factors	Likert Scales
Perceived ease of use and usability	7	Cognitive factors	Four-point Likert Scale (from *I strongly disagree* to *I strongly agree*)
Perceived usefulness	10	Cognitive factors	
Expectation about the iPad	9	Cognitive factors	
Anticipation of using the iPad	13	Cognitive factors	
Satisfaction when using the iPad	6	Affective factor	
Preference of using the iPad	8	Affective factor	
Knowledge about applications available on the iPad	18	Individual characteristic	Four-point Likert Scale (from *very low* to *very high*)
Self-efficacy	10	Individual characteristic	Six-point Guttman scale (from *Not at all confident* to *Totally confident*).
Self-reported use of an iPad	19	Use	Five-point Likert scale *(Never, Rarely, Sometimes, Often, Very often)*

Some items for the constructs considered in the study (self-efficacy, perceived ease of use and usability, perceived usefulness) were selected and adopted from previous research [23]. Satisfaction and preference came from Mang & Wardley [25], while others were added by the research team (for the constructs of, knowledge, anticipation, and expectation). Some of the items borrowed from previous studies were dropped or modified from the original research to better fit the context of the present study. Note that Cronbach Alpha coefficients reported in previous studies were very high, ranging from .87 to .93. The list of scale items before translation is presented in Table 3. In the online survey, students were also asked to identify their gender and age.

### Procedure

The survey was administered online, twice, to two cohorts of students. For both cohorts, surveys were administered during the second semester of the first year of the program (time 1) and during the second semesters of the second year of the program (time 2). Students received an email (at a preestablished date) requesting them to complete the survey on an online platform (Lime Survey). Email reminders were forwarded to non-responding students. From February 2017, the research committee partnered with some students to promote the survey in cohorts’ private social media group (i.e., Facebook) as well. Data were collected from Winter 2016 to Summer 2018. The consent form was embedded in the online survey, all participants gave informed consent electronically.

### Data analysis

Data from the two cohorts were combined. Given the small sample size as compared to the number of latent (constructs in the study) and observed (items of each construct) variables, we used the partial least squares structural equation modelling (PLS-SEM) via Smart-PLS version 3 to test measurement models (models that establish the relations between the constructs included in our survey) [26, 27]. This method allows multivariate analyses with small samples and is suitable for non-normal distribution data [26]. The use of PLS-SEM in research is increasing largely since 2015 [28].

Once measurement models are established based on evidence-informed or experience-informed hypothesis, they are statistically tested using four defined criteria, namely 1- construct validity, 2- internal consistency, 3- convergent validity, and 4- discriminant validity.

### Study hypotheses

The hypotheses underpinning this project are presented in Table 2. These hypotheses were based on previous findings of other authors on technology implementation in medical education [17–19], health professions education [29, 30], and authors that study the use of tablet in higher education [25] and were stated for first year (T1) and second year (T2). Note that some hypotheses were formulated by the team members, but were based on the literature of acceptance of technology. For example, Briz-Ponce et al. [17] found that anxiety (which is close to our concept of anticipation) influenced attitude (which in turn is supposed to influence behavioural intention). From this, we hypothesised that anticipation would influence the use of the iPad. Then, we proposed to explore at how perceived ease of use and usefulness and perceived usefulness could influence anticipation.

**Table 2:** Hypotheses tested for T1 and time T2

H_x_	Factors	Hypotheses	References supporting hypotheses
H_1_	Expectation -> perceives ease of use and usability	Students’ expectations have a positive effect on perceives ease of use and usability	Ashfaq et al. [31] Hong et al. [32 ] Liao et al. [33]
H_2_	Expectation -> perceived usefulness	Students’ expectations have a positive effect on perceived usefulness	Tam et al. [34]
H_3_	Anticipation -> use	Students’ anticipation has a negative effect on the use of an iPad	Team hypothesis
H_4_	Self-efficacy -> perceived ease of use and usability	Self-efficacy has a positive effect on perceived ease of use and usability	Venkatesh and Davis [22] Holden and Rada [23]
H_5_	Knowledge -> use	Students’ knowledge has a positive effect on the use of an iPad	Team hypothesis
H_6_	Perceives ease of use and usability -> anticipation	Perceived ease of use and usability have a negative effect on anticipation	Team hypothesis
H_7_	Perceives ease of use and usability -> preferences	Perceived ease of use and usability have a positive effect on preferences	Harmon [19]
H_8_	Perceives ease of use and usability -> satisfaction	Perceived ease of use and usability have a positive effect on satisfaction	Harmon [19]
H_9_	Perceives ease of use and usability -> use	Perceived ease of use and usability have a positive effect on the use of an iPad	Harmon [19]
H_10_	Perceives ease of use and usability -> perceived usefulness	Perceived ease of use and usability have a positive effect on perceived usefulness	Briz-Ponce and García-Peñalvo [18] Harmon [19]
H_11_	Preferences -> use	Students’ preferences have a positive effect on the use of an iPad	Harmon [19]
H_12_	Satisfaction -> use	Students’ satisfaction has a positive impact on the use of an iPad	Harmon [19]
H_13_	Perceived usefulness -> anticipation	Perceived usefulness has a negative effect on anticipation	Team hypotheses
H_14_	Perceived usefulness -> satisfaction	Perceived usefulness has a positive impact on satisfaction	Team hypotheses
H_15_	Perceived usefulness -> use	Perceived usefulness has a positive impact on the use of an iPad	Ducey and Coovert [30] Day-Black [29] Harmon [19]

## Results

### Descriptive statistics

For the first cohort, 68.6% of participants were women and mean age of participants was 20.5 years old (SD = 3.14). For the second cohorts, 59.1 % were women, and mean age of participants was 21.2 (SD = 3.35). We combined data from both cohorts for the analyses. Overall, 187 and 161 students respectively answered the questionnaires at T1 and T2.

As the method used in this study is PLS-SEM, as stated by Hair et al. [35], “the minimum sample size should be 10 times the maximum number of arrowheads pointing at the latent variable anywhere in the PLS path model” (p. 24). In our study, we have 14 arrows in our models, so 10 x 14 = 140. As 187 (44.8%) and 161 (38,5%) students respectively answered the questionnaires at T1 and T2, the samples should be large enough to conduct the PLS-SEM analyses.

### Measurement model

Because the constructs included in our survey were validated in previous studies, we have assessed the construct validity of their measurement scales with confirmatory factor analysis (CFA). More specifically, two measurement models (for T1 and T2) were assessed using the previously defined criteria (1- construct validity, 2- internal consistency, 3- convergent validity, and 4- discriminant validity).

After a preliminary analysis, some items were removed from the models (in T1 and T2) to meet these criteria. After removing these items, the models (in T1 and T2) were re-estimated again. All remaining items loaded significantly on their respective constructs, showing an adequate construct validity. Moreover, each item loaded higher on its construct than on any other construct. As for internal consistency, the values of Cronbach Alpha and composite reliability exceeded the threshold of 0.70, providing evidence for adequate internal consistency as suggested by Hair et al. [36]. Convergent validity is also achieved, as all the average variance extracted (AVE) exceeded .50 for T1 and T2. These results are reported in Table 3 and Table 4 for T1 and T2.

**Table 3:** List of scale items adapted to the context of the use of iPad in medical education setting, factor loadings, Cronbach Alpha, composite reliability and AVE

	T1				T2			
	Factor loadings	Cronbach Alpha	Composite reliability	AVE	Factor loadings	Cronbach Alpha	Composite reliability	AVE
***Use : Over the course of a week, how often do you do the following on an iPad while studying?***		*0.85*	*0.90*	*0.69*		*0.87*	*0.91*	*0.73*
Use_e: Consult reference books online	0.77				0.78			
Use_f : Highlight text	0.87				0.92			
Use_g : Annotate text	0.89				0.91			
Use_h : Take notes (in class, on the floor, etc.)	0.79				0.80			
***Perceived ease of use and Usability: In general, I find that …***						*0.91*	*0.93*	*0.73*
PU_b: … the iPad is easy to use.	0.85				0.88			
PU_c : … I can easily get the iPad to do what I want it to do.	0.87				0.83			
PU_d : … learning how to use the various iPad features is easy.	0.89				0.87			
PU_e : … the iPad features are practical and efficient.	0.88				0.85			
PU_g : … remembering how to do different things with the iPad is easy.	0.87				0.86			
***Self-efficacy : Using the iPad, I could do any task that was asked of me if …***		*0.92*	*0.93*	*0.61*		*0.93*	*0.93*	*0.61*
SE_a … there was no one around telling me what to do.	0.60				0.75			
SE_c … I had access to only the user manuals.	0.74				0.77			
SE_d … I could watch someone else do it first.	0.82				0.82			
SE_e … I could ask someone for help if I had a problem.	0.80				0.79			
SE_f … someone could help me just to get started.	0.79				0.89			
SE_g … I had a lot of time to complete the task.	0.84				0.76			
SE_h … I had only the textual help included with the feature (in the app).	0.83				0.80			
SE_i … someone clearly showed me how to do it first.	0.77				0.72			
SE_j … I had already completed a similar task using the same feature.	0.80				0.75			
***Knowledge : Indicate your level of knowledge of each of the features listed below.***		*0.85*	*0.90*	*0.69*		*0.88*	*0.92*	*0.74*
Knowledge_e: Consulting reference books online	0.73				0.75			
Knowledge_f : Highlighting text	0.91				0.93			
Knowledge_g : Annotating text	0.89				0.92			
Knowledge_h: Taking notes (in class, on the floor, etc.)	0.79				0.83			
***Satisfaction : I am satisfied …***		*0.87*	*0.90*	*0.65*		*0.86*	*0.90*	*0.64*
Satis_a: … with the iPad as a communications management tool.	0.78				0.75			
Satis_b: … with the iPad’s efficiency at processing and editing files and documents.	0.82				0.79			
Satis_d: … with the quality of the work (finished product) produced on the iPad.	0.78				0.78			
Satis_e: … with the unexpected possibilities of certain features.	0.80				0.82			
Satis_f: … with the way I use the iPad in my studies.	0.86				0.85			
***Preferences***		*0.82*	*0.89*	*0.73*		*0.80*	*0.88*	*0.71*
Preferences_a: I prefer to use the iPad as a technology to enhance my learning.	0.88				0.89			
Preferences_d: I feel good about the prospect of using an iPad throughout my studies.	0.89				0.87			
Preferences_e: I like the iPad itself.	0.78				0.76			
***Expectation : In using the iPad systematically in my studies, I expect …***		*0.72*	*0.84*	*0.64*		*0.78*	*0.87*	*0.69*
Expect_d: … that the iPad will contribute to my learning.	0.75				0.83			
Expect_g: … to use the iPad daily in my studies.	0.84				0.84			
Expect_h: … that the iPad will allow me to diversify my work methods.	0.79				0.83			
***Anticipation : In using the iPad systematically in my studies, I anticipate …***		*1.00*	*1.00*	*1.00*		*1.00*	*1.00*	*1.00*
Anticip_e : … not being able to do the same things I can do on paper.	1.00				1.00			
***Perceived usefulness : Using the iPad in my studies …***		*0.86*	*0.90*	*0.59*		*0.89*	*0.92*	*0.65*
PU_a: … could improve the efficiency of document and information management.	0.81				0.79			
PU_b: … could improve my professional performance overall.	0.82				0.83			
PU_c: … could make group sessions or meetings more productive.	0.73				0.80			
PU_d: … could make my study sessions more productive.	0.83				0.81			
PU_e: … could make me a better problem solver.	0.82				0.85			
PU_i: … would be beneficial to my future professional development.	0.59				0.75

**Table 4:** Discriminant validity [37] in T1 and T2

	T1									T2								
	1	2	3	4	5	6	7	8	9	1	2	3	4	5	6	7	8	9
1.Expectation	**0.80**									**0.83**								
2.Anticipation	-0.28	**1.00**								-0.33	**1.00**							
3.SE	0.20	-0.06	**0.78**							0.21	-0.04	**0.78**						
4.Knowledge	0.18	-0.17	0.32	**0.83**						0.27	-0.15	0.29	**0.86**					
5.PEOU	0.27	-0.23	0.44	0.43	**0.87**					0.29	-0.22	0.44	0.45	**0.86**				
6.Preferences	0.48	-0.40	0.22	0.30	0.48	**0.85**				0.51	-0.36	0.29	0.45	0.50	**0.84**			
7.Satisfaction	0.39	-0.38	0.21	0.40	0.57	0.72	**0.81**			0.53	-0.40	0.25	0.48	0.54	0.76	**0.80**		
8.Use	0.39	-0.33	0.06	0.35	0.24	0.46	0.42	**0.83**		0.38	-0.27	0.12	0.48	0.32	0.56	0.60	**0.85**	
9.PU	0.54	-0.32	0.19	0.30	0.38	0.62	0.59	0.42	**0.77**	0.56	-0.34	0.27	0.31	0.37	0.67	0.67	0.56	**0.81**

### Hypotheses testing

**Fig. 1:**
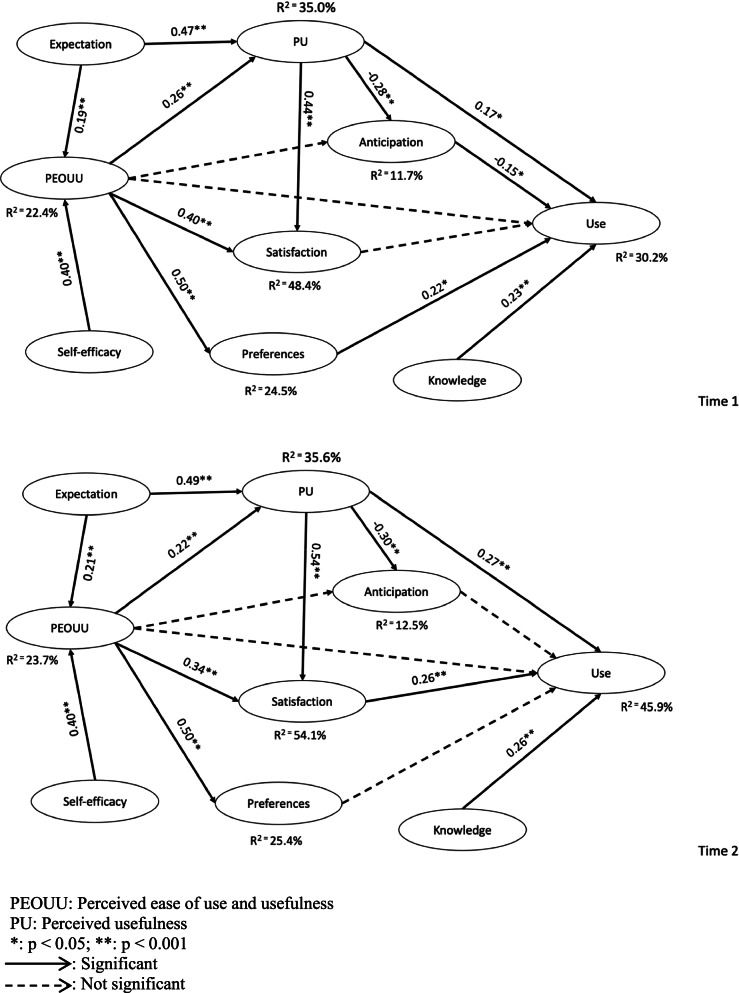
Overall model results for T1 and T2

We tested our hypotheses using a unilateral significance level of 0.05 because of the direction they were given during formulation of the hypotheses following the Theoretical Framework. The *t* tests are thus one-tailed and the *p* values detailed in Table 1 according to these hypotheses are adjusted for unidirectionality with a *p* value smaller than 0.05 representing a statistical significance. Table 5 details the summary of path coefficients, *t*-values, *p*-values and R^2^ values in T1 and T2. These results are also presented in Fig. 1.

**Table 5:** Summary of results according to hypotheses in T1 and T2

		T1 (*n* = 187)				T2 (*n* = 161)			
		*β*	*t*	*p*		*β*	*t*	*p*	
H_1_	Expectation -> Perceives ease of use and Usability	0.19	2.75	0.0030	Yes	0.21	2.92	0.0020	Yes
H_2_	Expectation -> Perceived usefulness	0.47	5.81	0.0000	Yes	0.49	7.16	0.0000	Yes
H_3_	Anticipation -> Use	-0.15	1.75	0.0410	Yes	-0.02	0.32	0.3740	No
H_4_	Self-efficacy -> Perceived ease of use and Usability	0.40	6.44	0.0000	Yes	0.40	6.17	0.0000	Yes
H_5_	Knowledge -> Use	0.23	2.64	0.0040	Yes	0.26	3.86	0.0000	Yes
H_6_	Perceives ease of use and Usability -> Anticipation	-0.12	1.52	0.0640	No	-0.11	1.25	0.1070	No
H_7_	Perceives ease of use and Usability -> Preferences	0.50	7.17	0.0000	Yes	0.50	7.33	0.0000	Yes
H_8_	Perceives ease of use and Usability -> Satisfaction	0.40	6.01	0.0000	Yes	0.34	5.77	0.0000	Yes
H_9_	Perceives ease of use and Usability -> Use	-0.11	1.05	0.1480	No	-0.08	0.88	0.1890	No
H_10_	Perceives ease of use and Usability -> Perceived usefulness	0.26	3.12	0.0010	Yes	0.22	2.88	0.0020	Yes
H_11_	Preferences -> Use	0.22	2.02	0.0220	Yes	0.09	0.81	0.2090	No
H_12_	Satisfaction -> Use	0.07	0.65	0.2570	No	0.26	2.56	0.0050	Yes
H_13_	Perceived usefulness -> Anticipation	-0.28	3.92	0.0000	Yes	-0.30	3.22	0.0010	Yes
H_14_	Perceived usefulness -> Satisfaction	0.44	6.43	0.0000	Yes	0.54	10.95	0.0000	Yes
H_15_	Perceived usefulness -> Use	0.17	1.89	0.0300	Yes	0.27	2.91	0.0020	Yes
	R^2^ Anticipation	11.7%				12.5%			
	R^2^ PEOUU	22.4%				23.7%			
	R^2^ Satisfaction	48.4%				54.1%			
	R^2^ Use	30.2%				45.9%			
	R^2^ PU	35.0%				35.6%

In T1, 12 paths were statistically significant, which means that 12 out of 15 hypotheses were confirmed. More specifically, the strongest driver of the use of iPads was knowledge followed by preferences, and perceived usefulness, and then negatively influenced by anticipation. The other indirect effects observed were expectation being a strong driver of perceived usefulness, and to a lesser extend of perceived ease of use and usability. Self-efficacy influenced perceived ease of use and usability. Then, perceived ease of use and usability was an important driver of preferences, satisfaction, and perceived usefulness. Perceived usefulness in return influenced positively satisfaction and negatively anticipation. The R^2^ coefficient of determination indicates that 30.2% of the variability in iPad use could be explained by the structural model. In T2, 11 paths were significant, and 11 out of 15 hypotheses were confirmed. The main variables explaining the use of iPad are: perceived usefulness, knowledge and satisfaction. The other indirect effects are the same as in T1. The R^2^ indicates that 45.9% of the variability in iPad use could be explained by the structural model. Moreover, some paths that were significant in T1 are not significant any more in T2 and vice-versa.

## Discussion

The objectives of this study were two-fold: first to identify factors that influenced undergraduate medical students’ use of an iPad at two different moments in the preclinical curriculum, and second to study how those factors evolved over the year. The main factors influencing the use of the iPad varied between our measurement times (year in the undergraduate medical education program). For example, satisfaction did not influence the use of an iPad during the first year but did in the second year. Preferences and anticipation influenced the use of the iPad during the first year but did not in the second year.

### Differences between T1 and T2

Satisfaction did not influence the use of an iPad during the first year but did in the second year. Students may have acquired experience using the iPad, thus becoming more (or less) satisfied with its use and using it consequently. This result suggests that while the novelty of the iPad wears off, satisfaction can develop over time with exposure to the technology features (such as being satisfied with the iPad for communication, or to treat and edit documents, or to be satisfied of the results of a work on the iPad).

Preferences influenced positively the use of the iPad only during the first year; this might be due to the novelty of the iPad in the program. In other words, students reported wanting to use it, because it was something new. When the novelty wears off by the second year, students may realize they prefer using other devices, such as a computer, to do their academic work. Similarly, Harmon [19] observed no influence of perceived enjoyment of an application by medical students on their intention to use it.

Anticipation negatively influenced the use of the iPad in the first year of use and was measured by the item “anticipating not being able to do with the iPad what I do on paper.” Briz-Ponce et al. [17] had a similar construct to what we called anticipation, that is anxiety, that measured the apprehension of using the technology. In their study, although they did not consider the direct relationship of anxiety on the behavior, they found a negative relationship between this factor with attitude. In our study, negative anticipation decreased the use of technology, which might simply be explained by the fear of the unknown and again supports the importance of appropriate training offered to students. This hypothesis is further supported by the fact that the negative effect of anticipation disappeared in the second year of using the iPad.

### Other factors that increased the use of the iPad

Knowledge about the iPad was an important driver of using it; this was independent of the progression in the curriculum. Intuitively these results make sense: one needs to know the applications and their potential to use them. In our context more specifically, knowing how to use books in an application, as well as annotation and notes features, were drivers of iPad use. This observation highlights the importance of appropriately training students to the features and opportunities afforded when using the iPad. Perceived usefulness also influenced iPad use at both data collection times. This is a usual hypothesis of the TAM model [38], and a hypothesis that also is supported in other studies conducted in the health professions education [19, 20, 29, 30]. In other words, one needs to believe in the usefulness of technology to use it.

Some variables had an *indirect influence* on the use of the iPad. Perceived ease of use and usability influenced the use through satisfaction in the T2 model. Self-efficacy is also related to perceived ease of use and usability as a driver of it, which was proposed by Holden et Rada [23]. We also observed that perceived usefulness influenced positively students’ satisfaction with the iPad. While satisfaction may be difficult to influence directly, it may be influenced through perceived ease of use and usability, the same can be done to increase iPad use through preferences. Promoting self-efficacy by providing a technology known from students may contribute indirectly, through perceived ease of use and usability, and then perceived usefulness or satisfaction, to the use of the technology being implemented.

We observed that perceived usefulness influenced anticipation, that is, as the perceived usefulness increased, the negative anticipation diminished. Similarly, expectation was a driver of perceived ease of use and usability and perceived usefulness. In our survey, this concept revolved around student’s expectation of how using the iPad will contribute to his learning, student’s expectation about using the iPad daily in his study, and expectation about the iPad allowing to diversify study methods. This means that increased expectations about the iPad led to an increase in perceived ease of use and usability. Thus, intervening at the level of expectation might be important when introducing technology in preclinical settings by again showing what one can do with the technology, and providing enough supports so the student can have great expectation about using the technology during his study.

What can be gleaned from our results is that if a program wants to encourage the use of a mobile device by its students, it should offer training to maximize knowledge about said device and how to utilize it to its full potential. Administrators and educators may also want to explicitly discuss and demonstrate to students the usefulness and utility of a technology if they aim to increase student’s positive affect about the technology, and consequently, its use. Without such interventions as to make explicit potential usefulness and utility, in addition to functions, students are left at their own device to identify how the technology can contribute to their development.

### Strengths and limitations

To our knowledge, this was the first study to document, longitudinally, factors that influence the use of iPad in a preclinical undergraduate medical education curriculum. This study has strong theoretical underpinning, increasing the potential impact and generalizability of our findings to other similar contexts, where medical students may need an extra help to navigate through a plethora of knowledge that is beyond simple memorization. This study also adds knowledge on mobile technologies acceptance in the context of higher education in North America [16]. In addition, we observed good model fit, also increasing the range of our findings and contributing to the refinement of the TAM by having explored other potential constructs of interest to explain the use of a technology in a learning setting.

Our study has limitations. We cannot exclude the probability that only motivated or positive students participated in our study since we employed a convenience sample. In addition, we had an overall small sample, however comparable to samples used in other studies in the field [17, 18]. Replication studies would add value to our results. We also kept the variable “anticipation” in the model even though we only had one item remaining. A future study should further explore this factor and its items to make it stronger and validate its importance or not in the model. We only looked at students’ perspectives, while faculty could play an important role in the implementation of mobile technology. Studying faculty acceptance of mobile technology would be essential to guarantee a successful implementation. Future studies could investigate faculty acceptance of mobile technology. Finally, while our undergraduate medical education program decided, at that time, to adopt the iPad, there were many other mobile technologies accessible to our participants and we cannot establish if this had any influence on their responses to the survey. A future study could consider the influence of these technologies on acceptance and use of iPad devices by undergraduate medical students.

## Conclusion

It is important to help students acquire knowledge, and nowadays, it is even more important to help students develop the skills to access the ever-growing knowledge they need to practice their profession. The use of the TAM in this study allowed us to identify factors that contribute to the use of iPads in an undergraduate medical education preclinical setting and help to strengthen its implementation. We found in this study that strategies to better the use of mobile technologies need to be adapted to the learner progression in the curriculum. More specifically, one could aim for a proper introduction to technologies that emphasized its utility and usefulness, with a proper training so students perceived its ease of use, and encourage preferences for the said technology, whilst trying to respond to students’ anticipation for more junior students. When dealing with students that have more experience in the curriculum, more energy should be put on satisfaction and less on anticipation and preferences.

## Abbreviations

PLS-SEM: Partial least squares structural equation modelling
TAM: Technology Acceptance Model
FMSS: Faculté de médecine et des sciences de la santé
CFA: Confirmatory factor analysis
AVE: Average variance extracted

## Footnotes

1 https://apps.apple.com/ca/app/medscape/id321367289?form=MY01SV&OCID=MY01SV

2 https://apps.apple.com/ca/app/medcalx/id1041464932

## References

[1] Densen P (2011). Challenges and opportunities facing medical education. Trans Am Clin Climatol Assoc.

[2] Alper BS, Hand JA, Elliott SG, Kinkade S, Hauan MJ, Onion DK (2004). How much effort is needed to keep up with the literature relevant for primary care?. J Med Libr Assoc.

[3] Mallett S, Clarke M (2003). How many Cochrane reviews are needed to cover existing evidence on the effects of healthcare interventions?. BMJ Evid-Based Med.

[4] George P, Dumenco L, Dollase R, Taylor JS, Wald HS, Reis SP (2013). Introducing technology into medical education: two pilot studies. Patient Educ Couns.

[5] Mehta N, Shamdas M (2015). Tablets for tomorrow’s doctors. Clin Teach.

[6] Youm J, Wiechmann W (2015). Medical student use of the iPad in the clerkship curriculum. Clin Teach.

[7] Boruff JT, Storie D (2014). Mobile devices in medicine: a survey of how medical students, residents, and faculty use smartphones and other mobile devices to find information. J Med Libr Assoc JMLA.

[8] Ellaway RH, Fink P, Graves L, Campbell A (2014). Left to their own devices: medical learners’ use of mobile technologies. Med Teach..

[9] George P, Dumenco L, Doyle R, Dollase R (2013). Incorporating iPads into a preclinical curriculum: A pilot study. Med Teach.

[10] Byrne-Davis L, Dexter H, Hart J, Cappelli T, Byrne G, Sampson I, Mooney J, Lumsden C. Just-in-time research: a call to arms for research into mobile technologies in higher education. Research in Learning Technology [Internet]. 2015;230. [cited 2022 Feb 6]. Available from: https://journal.alt.ac.uk/index.php/rlt/article/view/1600.

[11] Robinson R (2015). Spectrum of tablet computer use by medical students and residents at an academic medical center. PeerJ..

[12] Omori JS, Wong VS, Nishimura S (2013). Medical school hotline: Enhancing problem-based learning with technology: the introduction of iPads into the John A. Burns School of Medicine Curriculum. Hawaii J Med. Public Health.

[13] Davies BS, Rafique J, Vincent TR, Fairclough J, Packer MH, Vincent R (2012). Mobile Medical Education (MoMEd)-how mobile information resources contribute to learning for undergraduate clinical students-a mixed methods study. BMC Med Educ.

[14] Wallace S, Clark M, White J. 'It's on my iPhone': attitudes to the use of mobile computing devices in medical education, a mixed-methods study. BMJ Open. 2012;2(4):e001099. 10.1136/bmjopen-2012-001099. 10.1136/bmjopen-2012-001099 PMC3432838 22923627

[15] Deutsch K, Gaines JK, Hill JR, Nuss MA (2016). iPad experience during clinical rotations from seven medical schools in the United States: lessons learned. Med Teach.

[16] Al-Emran M, Mezhuyev V, Kamaludin A. Technology Acceptance Model in M-learning context: A systematic review. Comput Educ 1 oct 2018;125:389-412.

[17] Briz-Ponce L, Pereira A, Carvalho L, Juanes-Méndez JA, García-Peñalvo FJ (2017). Learning with mobile technologies – Students’ behavior. Comput Hum Behav.

[18] Briz-Ponce L, García-Peñalvo FJ (2015). An Empirical Assessment of a Technology Acceptance Model for Apps in Medical Education. J Med Syst.

[19] Harmon DJ. User Acceptance of a Novel Anatomical Sciences Mobile App for Medical Education - An Extension of the Technology Acceptance Model [Internet]. [Columbus, OH]: The Ohio State University; 2015. Disponible sur: http://rave.ohiolink.edu/etdc/view?acc_num=osu1437408234

[20] Hart J, Sutcliffe A (2019). Is it all about the Apps or the Device?: User experience and technology acceptance among iPad users. Int J Hum-Comput Stud.

[21] Davis FD (1993). User acceptance of information technology: system characteristics, user perceptions and behavioral impacts. Int J Man-Mach Stud.

[22] Venkatesh V, Davis FD (2000). A theoretical extension of the technology acceptance model: Four longitudinal field studies. Manag Sci.

[23] Holden H, Rada R (2011). Understanding the influence of perceived usability and technology self-efficacy on teachers’ technology acceptance. J Res Technol Educ.

[24] Townsend AM, Demarie SM, Hendrickson AR (2001). Desktop video conferencing in virtual workgroups: anticipation, system evaluation and performance. Inf Syst J.

[25] Mang C, Wardley L. Student perceptions of using tablet technology in post-secondary classes / Perceptions des étudiants quant à l’utilisation des tablettes électroniques dans les classes universitaires. Can J Learn Technol Rev Can L’apprentissage Technol [Internet]. 2013; [cited 2001 Feb 10];39(4). Available from: https://www.learntechlib.org/p/130196/.

[26] Chin WW (2001). PLS-Graph user’s guide. CT Bauer Coll Bus Univ Houst USA.

[27] Gefen D, Straub D (2005). A practical guide to factorial validity using PLS-Graph: Tutorial and annotated example. Commun Assoc Inf Syst.

[28] Ghasemy M, Teeroovengadum V, Becker J-M, Ringle CM (2020). This fast car can move faster: a review of PLS-SEM application in higher education research. High Educ.

[29] Day-Black CY. Predictors of nursing faculty acceptance of mobile information technology in baccalaureate nursing education [thesis]. Tuscaloosa (AL: University of Alabama Libraries; 2017 [cited 2021 Feb 10]. Available from: http://ir.ua.edu/handle/123456789/3218

[30] Ducey AJ, Coovert MD (2016). Predicting tablet computer use: An extended Technology Acceptance Model for physicians. Health Policy Technol.

[31] Ashfaq M, Yun J, Waheed A, Khan MS, Farrukh M. Customers’ Expectation, Satisfaction, and Repurchase Intention of Used Products Online: Empirical Evidence From China. SAGE Open. 2019;9(2):2158244019846212.

[32] Hong S, Thong JY, Tam KY. Understanding continued information technology usage behavior: A comparison of three models in the context of mobile internet. Decis Support Syst. 2006;42(3):1819‑34.

[33] Liao C, Chen J-L, Yen DC. Theory of planning behavior (TPB) and customer satisfaction in the continued use of e-service: An integrated model. Comput Hum Behav. 2007;23(6):2804‑22.

[34] Tam C, Santos D, Oliveira T. Exploring the influential factors of continuance intention to use mobile Apps: Extending the expectation confirmation model. Inf Syst Front. 2020;22(1):243‑57.

[35] Hair JFJ, Hult GTM, Ringle C, Sarstedt M. A primer on partial least squares structural equation modeling (PLSSEM). 3 ed. Los Angeles, CA: SAGE Publications; 2021.

[36] Hair JFJ, Hult GTM, Ringle C, Sarstedt M. A primer on partial least squares structural equation modeling (PLSSEM). 2 ed. Los Angeles, CA: SAGE Publications; 2017.

[37] Fornell C, Larcker DF. Evaluating structural equation models with unobservable variables and measurement error. J Mark Res. 1981;18(1):39‑50.

[38] Davis FD, Bagozzi RP, Warshaw PR. User acceptance of computer technology: A comparison of two theoretical models. Manag Sci. 1989;35(8):982‑1003.

